# Potential Implications of Quercetin in Autoimmune Diseases

**DOI:** 10.3389/fimmu.2021.689044

**Published:** 2021-06-23

**Authors:** Pan Shen, Weiji Lin, Xuan Deng, Xin Ba, Liang Han, Zhe Chen, Kai Qin, Ying Huang, Shenghao Tu

**Affiliations:** ^1^ Department of Integrated Chinese Traditional and Western Medicine, Tongji Hospital, Tongji Medical College of Huazhong University of Science and Technology, Wuhan, China; ^2^ Department of Nephrology, Zhongnan Hospital of Wuhan University, Wuhan, China

**Keywords:** autoimmune diseases, quercetin, rheumatoid arthritis, inflammation, oxidative stress

## Abstract

Autoimmune diseases are a worldwide health problem with growing rates of morbidity, and are characterized by breakdown and dysregulation of the immune system. Although their etiology and pathogenesis remain unclear, the application of dietary supplements is gradually increasing in patients with autoimmune diseases, mainly due to their positive effects, relatively safety, and low cost. Quercetin is a natural flavonoid that is widely present in fruits, herbs, and vegetables. It has been shown to have a wide range of beneficial effects and biological activities, including anti-inflammation, anti-oxidation, and neuroprotection. In several recent studies quercetin has reportedly attenuated rheumatoid arthritis, inflammatory bowel disease, multiple sclerosis, and systemic lupus erythematosus in humans or animal models. This review summarizes the evidence for the pharmacological application of quercetin for autoimmune diseases, which supports the view that quercetin may be useful for their prevention and treatment.

## Introduction

Autoimmune diseases are a heterogeneous cluster of disorders characterized by systemic syndromes, in which autoreactive adaptive immune responses contribute to immune-mediated damage to cells and organs (1). The pathogenesis and etiology of these diseases is not completely clear, but a complex interplay of genetic risk, environmental factors, mental factors, and stochastic events is considered to disrupt the balance of immunological tolerance and autoimmunity (2, 3). The polyphenol quercetin (3,3′,4′,5,7-pentahydroxyflavone) is a dietary flavonoid that is commonly found in vegetables (asparagus), fruits (apples, capers, chokeberries, cranberries), and medicinal herbs including lovage, dill, cilantro, and radish leaves (4–6). Many previous studies have shown that appropriate doses of quercetin have a variety of biological activities and neuroprotective, anti-allergic, anti-oxidant, anti‐inflammatory, immunomodulatory, anti-microbial, and anti-tumor effects (7–12). Various ongoing scientific studies are investigating the use of natural products to develop drugs to treat autoimmune diseases. Quercetin may be useful as a dietary supplement to prevent or treat autoimmune diseases.

In the present manuscript we will summarize the protective effects of quercetin in multiple autoimmune diseases, and evaluate the molecular mechanisms of quercetin that can modulate immune responses.

## Biological Activity and Functions of Quercetin

In dietary supplements, daily doses of quercetin are usually in the range of 1 to 250 mg (13). After ingestion quercetin can combine with salivary proteins to form soluble protein-quercetin binary aggregates (14). Upon arrival in the small intestine quercetin is deglycosylated by lactate phlorizin hydrolase to produce quercetin aglycon. In the cecum and colon, quercetin glucosides can be directly taken in *via* sodium-dependent glucose transporter-1, or secreted into the lumen *via* multidrug resistance protein 2 (15). Quercetin can be absorbed into epithelial cells *via* lipophilicity-dependent dispersion. Before entering the circulatory system most of the quercetin ingested from the intestinal cavity is converted into conjugated metabolites (16). Approximately 60%–81% of quercetin is channeled to the liver *via* the epithelium, where it is metabolized and converted into a bioavailable form (17). Most quercetin and its metabolites are excreted *via* the intestines, but small amounts are excreted by the kidneys in the urine (18). Recent studies suggest that gut microbiota participate in the production of glycosidases and enzymes that convert quercetin into more readily absorbable molecules (19, 20). Quercetin can evidently be metabolized as glucuronidated, methylated, and sulphated derivatives such as homoprocatechuic acid, protocatechuic acid, and 4-hydroxybenzoic acid (21).

Quercetin reportedly exerts powerful anti-inflammatory effects, mainly *via* inhibition of cytokine production, reduction of cyclooxygenase and lipoxygenase expression, and maintaining the stability of mast cells (22–24). It can reportedly reduce *Streptococcus suis*-induced inflammation by inhibiting the activation of p38 mitogen-activated protein kinase, extracellular signal-related kinases (ERK1/2), and nuclear factor kappa B (NF-κB). It can also reduce the production of pro-inflammatory cytokines such as tumor necrosis factor (TNF)-α, interleukin (IL)-1β, and IL-6 (25). In one study, treatment with quercetin at a dose of 25 mg/kg in diabetic rats reduced the production of prostaglandin E-2, IL-1β, and leukotriene B-4, promoting wound healing in rats (26). Similarly quercetin inhibited the production of TNF-α, IL-6, and IL-1 in lipopolysaccharide-activated human mononuclear U937 cells  (27). An indomethacin-mediated increase in myeloperoxidase activity in stomach and ileum tissues, as well as activation of NF-κB and the production of IL-8 in Caco-2 cells were also inhibited by quercetin (10 μg/mL) (28). In lipopolysaccharide-treated RAW264.7 cells, quercetin inhibited the production of TNF-α and nitric oxide synthase (iNOS), and the phosphorylation and activation of Jun N-terminal kinase/stress-activated protein kinase. Additionally, quercetin suppressed NF-κB translocation, AP-1, and NF-κB-DNA-binding and reporter gene transcription (29). It can reportedly protect human umbilical vein endothelial cells from inflammation induced by H_2_O_2_, which is mediated by downregulation of vascular cell adhesion molecule 1 and CD80 (30). As well as inhibiting pro-inflammatory cytokines such as TNF-α, IL-1β, and IL-6, quercetin also promotes the secretion of anti-inflammatory cytokines such as IL-10 (31).

The excellent anti-oxidant activity of quercetin is mainly exerted *via* effects on the activity of glutathione, enzymes, and reactive oxygen species (ROS), and regulating signal transduction pathways such as heme oxygenase 1/nuclear factor erythroid 2-related factor (Nrf2), mitogen-activated protein kinase, toll-like receptor 4/phosphatidylinositol-3-kinase, and 5’ adenosine monophosphate-activated protein kinase (32). Quercetin can reduce high-valent iron, thereby inhibiting lipid oxidation and quenching ROS, assisting in suppressing inflammation and preventing related diseases (33). Kalantari et al. (34) reported that quercetin significantly alleviated liver damage in mice by suppressing free radicals and upregulating the levels of antioxidant enzymes including glutathione peroxidase, superoxide dismutase, and catalase. In another study, a quercetin-coated nanocellulose matrix had strong antioxidant activity (35). Bai et al. (36) formulated composite films of carboxymethyl chitosan-quercetin that exhibited anti-oxidant capacity in a sustained manner in aqueous and lipid foods. Lastly, it has been reported that quercetin has neuroprotective effects and anti-cancer activity.

Quercetin has undergone clinical trials, and to date no significant toxicity or side effects have been observed in humans. Inhibitory effects of quercetin on inflammation have been observed in clinical studies, as it has the capacity to suppress multiple types of cancer (37). More clinical studies should be conducted to confirm the effects of quercetin on autoimmune diseases, and better characterize the potential mechanisms of these beneficial effects.

## Quercetin and Autoimmune Diseases

### Rheumatoid Arthritis

Rheumatoid arthritis (RA) is a systemic chronic autoinflammatory disease. The main clinical symptoms are synovitis and progressive destruction of multiple joints, which can lead to joint deformity and dysfunction. It can also cause multi-system damage, and in severe cases premature death. In a randomized, double-blind, placebo-controlled clinical study, quercetin significantly alleviated morning stiffness and pain in RA patients (38). In that study, DAS 28 and HAQ scores of RA patients in a quercetin group where lower than those in a placebo group. In *in vivo* studies (39–41), quercetin has reduced arthritic scores and improved symptoms significantly in RA mice *via* inhibiting neutrophil infiltration and reducing levels of pro-inflammatory cytokines such as interferon γ, TNF-α, monocyte chemotactic protein 1, IL-6, and IL-17. It also inhibited neutrophil extracellular trap formation by suppressing autophagy (42). In another study, in a murine arthritis model, quercetin treatment reduced IL-1β and TNF-α release, and inhibited levels of pre-pro-endothelin 1 and cyclooxygenase 2 mRNA by inhibiting the activation of NF-κB and heme oxygenase 1/Nrf2 signaling (43). Yang et al. (44) found that quercetin attenuated collagen-induced arthritis by regulating the Th17/Treg balance, upregulating the expression of heme oxygenase 1, and inhibiting the activation of NLRP3 inflammasome. Saccol et al. (45) reported that quercetin protected against joint damage by reversing the deleterious effects of complete Freund’s adjuvant‐induced rat arthritis. In that study, the protective effects were closely associated with reductions in aspartate aminotransferase, ROS, and thiobarbituric acid‐reactive substances, an increase in catalase activity, and reductions in DNA damage and fragmentation of double‐strand DNA.

The aberrant migration, proliferation, and invasion of fibroblast-like synoviocytes (FLSs) are considered to be major parts of the etiopathogenesis of RA. *In vitro*, quercetin significantly inhibited the migration and invasion of FLSs, and reduced the levels of F-actin. It also increased the level of miR-146a, but inhibited the expression of GATA transcription factor 6 in FLSs (46). Kim et al. (47) reported that quercetin reduced this osteoclast differentiation when CD14^+^ monocytes were cultured with IL-17-prestimulated RA-FLSs or Th17 cells, and it reduced IL-17-induced receptor activator of nuclear factor kappa-Β ligand (RANKL) levels in RA-FLSs, and inhibited the activation of the mammalian target of rapamycin. Many studies indicate that quercetin can inhibit the generation of osteoclast-like cells, bone resorption depression, and F-actin ring formation in RAW264.7 cells, bone-marrow macrophages, and human peripheral-blood mononuclear cells pre-treated with RANKL or macrophage colony-stimulating factor (48–52). These results suggest that quercetin may play a role in protection against arthritic bone destruction.

RA affects extra-articular tissues and organs, including blood vessels, the nervous and gastrointestinal systems, heart, lung, and kidneys. In an adjuvant-induced arthritis model established in rats, Piovezana Bossolani et al. (53) detected neurodegenerative effects on the enteric nervous system of the jejunum, and inflammation in the intestinal mucosa. Treatment with quercetin alone reversed the aforementioned neurodegenerative effects to an extent, possibly due to its anti-inflammatory, anti-oxidant, anti-arthritic, and neuroprotective capacities.

### Inflammatory Bowel Disease

Inflammatory bowel disease (IBD) encompasses a group of complicated and multifactorial polygenic conditions, mainly comprising ulcerative colitis and Crohn’s disease. It is characterized by chronic, progressive, and relapsing inflammation in the gastrointestinal tract, which increases the risk of colitis-associated cancer. Quercetin has been shown to be a potent anti-inflammatory compound in a variety of *in vitro* and *in vivo* bioassay models, but oral quercetin has not exhibited the desired effects in a colitis model (54, 55). This could be partly because it is absorbed in the upper gastrointestinal tract and thus does not reach the lower gastrointestinal tract. The rapid metabolism of quercetin is disadvantageous with respect to treating IBD, unless the flavonoid is introduced in its glycosylated form, the most common of which is the compound rutin. Only approximately 10% of orally ingested rutin is generally able to pass epithelial cells, the rest is converted to sulphate and glucuronide derivatives by the gut microbiota to generate quercetin (56). Although rutin may have direct effects in the treatment of IBD, it is reasonable to surmise that its pharmacological activity and effects are exerted *via* conversion to the bioactive aglycone, quercetin (57). Rutin is hydrolyzed by the gut bacteria in the colon to synthesize quercetin, so it may act as a prodrug with quercetin being the active component. Rutin has been shown to have potent effects *in vivo* (58).

In a trinitrobenzenesulfonic acid-induced inflammatory colitis rat model, oral administration of 10 or 25 mg/kg of rutin reduced colonic damage (59). In a dextran sulfate sodium (DSS)-induced colitis mouse model, rutin had a positive role in controlling colonic inflammation and disease progression, as well as in the reduction of nitric oxide, iNOS, cyclooxygenase 2, and prostaglandin E2 (60). Mascaraque et al. (61) reported that rutin (57 mg/kg/day orally) significantly improved CD4^+^ CD62L^+^ T cell transfer colitis in mice, but quercetin had no obvious effects. Rutin has also exhibited promising results in the rat model of acetic acid-induced colitis, in which pre-treatment (25 and 100 mg/kg) resulted in significant improvements in inflammatory indicators (62). In recent studies, quercetin alleviated DSS-induced colitis by strengthening intestinal integrity and liver antioxidant capacity, regulated ERK1/2-FKBP and RXR-STAT3 pathways (63), increased levels of glutathione in serum, and inhibited oxidative stress in a Caco-2 cell model induced by H_2_O_2_ (64). Dietary supplementation with 30 mg/kg of quercetin exerts therapeutic effects in a *Citrobacter rodentium*-induced colitis model in C57BL/6 mice, in part due to its ability to attenuate pro-inflammatory cytokines and regulate gut microbiota (65). Ju et al. (66) reported that quercetin could ameliorate DSS-induced colitis, and speculated that this was most likely due to modulating the anti-inflammatory effects of macrophages *via* the heme oxygenase 1-dependent pathway.

Some evidence suggests that glycosylation of quercetin as demonstrated by rutin is an important structural feature of flavonoids with respect to their efficacy against IBD. The deglycosylation of flavonoids in the small intestine is induced by epithelial β-glucosidases and colonic microflora, resulting in the production of bioactive aglycones such as quercetin (67). Quercetin and its glycosides—which are common in the blood in conjugated products—need be designed such that post-absorption they release active quercetin with a specific pattern in the colon. Several experiments of formulations have been conducted in recent years to address this problem and enhance the pharmacological efficacy of quercetin. The oral administration of quercetin-loaded microcapsules generated with a pectin/casein polymer attenuated macroscopic damage and edema, reduced neutrophil recruitment, and reduced levels of IL-1β and IL-33 in the colon tissues of mice with acetic acid-induced colitis (68). Shen et al. (69) designed a dual-responsive prodrug micelle consisting of quercetin and the biocompatible glycol chitosan, which tended to accumulate in sites of intestinal inflammation, and exhibited better therapeutic efficacy than the free drugs quercetin and mesalazine in a mouse colitis model.

Overall, available evidence suggests the efficacy of orally ingested glycosylated forms of quercetin such as rutin, or quercetin delivered *via* drug carriers for IBS. Sulfasalazine, a common aminosalicylate drug, also functions *via* interaction with colonic microbiota resulting in the release of active moieties at the IBD site. Unfortunately, however, the non-pharmacologically active fragment of sulfadiazine that is cleaved during this process has systemic side effects. The main drugs currently used to treat IBD such as 5-aminosalicylates, corticosteroids, immune-modifying agents, and biologic agents have exhibited disadvantages including loss of efficacy, substantial costs, and unavailability of formulations designed for oral administration. Quercetin is one of the most abundant natural flavonoids, and has promising therapeutic potential for the treatment of IBD. Oral rutin leading to the release of the active biomolecule quercetin at the site of inflammation may be an effective therapy for IBD.

### Multiple Sclerosis

Multiple sclerosis (MS) is an autoimmune inflammatory disease of the central nervous system characterized by extensive demyelination and neurodegeneration due to glia activation, oligodendrocyte death, and axon depletion. In an *in vitro* study conducted using peripheral blood mononuclear cells from MS patients, treatment with quercetin reduced their proliferation, and modulated levels of IL-1β, matrix metalloproteinase 9, and TNF-α in cell culture supernatants (70). Chronic microglia activation can result in the production of inflammatory and neurotoxic mediators including nitric oxide, iNOS, and ROS, which are closely associated with the pathogenesis and development of MS (71). The compound 3’-O-(3-chloropivaloyl) quercetin reduced the expression of iNOS in lipopolysaccharide-activated BV-2 microglia and inhibited the activation of NF-κB (72). In a mouse study utilizing experimental allergic encephalomyelitis, a Th1 cell-mediated inflammatory demyelinating disorder that is the most commonly used autoimmune model of MS, treatment with quercetin inhibited the IL-12-induced activation of JAK2, TYK2, STAT3, and STAT4, as well as Th1 differentiation (73). Mast cells are involved in inflammatory processes and allergic responses in which immunologic stimulation causes the production of inflammatory mediators. It has been suggested that mast cells are the immune gate of the brain, and are likely associated with neuropathologic processes including MS (74). Quercetin has been associated with reductions in the release of tryptase and IL-6, and inhibition of histidine decarboxylase mRNA from human mast cells  (75). Quercetin may be useful in the complementary treatment for MS.

### Other Autoimmune Diseases

Systemic lupus erythematosus is a disorder characterized by immune-mediated inflammation and over-production of autoantibodies. Quercitrin attenuated the symptoms of lupus nephritis in a systemic lupus erythematosus mouse model, partly *via* inhibition of CD4^+^ T cell activation and inflammatory reactions by macrophages (76). Graves’ disease is the most common autoimmune thyroid disorder associated with hyperthyroidism. Quercetin can reportedly inhibit DNA damage by modulating oxidative stress in lymphocytes from Graves’ disease patients *in vitro* (77). Atopic dermatitis is an autoimmune and inflammatory skin disease characterized by skin lesions exhibiting infiltration by mast cells, eosinophils, and macrophages. In a mouse model of atopic dermatitis, treatment with quercetin reduced the concentrations of TNF-α, CCL17, IL-4, IL-6, and IFN-γ in skin tissues (78), downregulated HMGB1, RAGE, p-NF-κB, and ERK1/2 levels, and upregulated Nrf2 (79, 80).

## Conclusion

**Figure 1:**
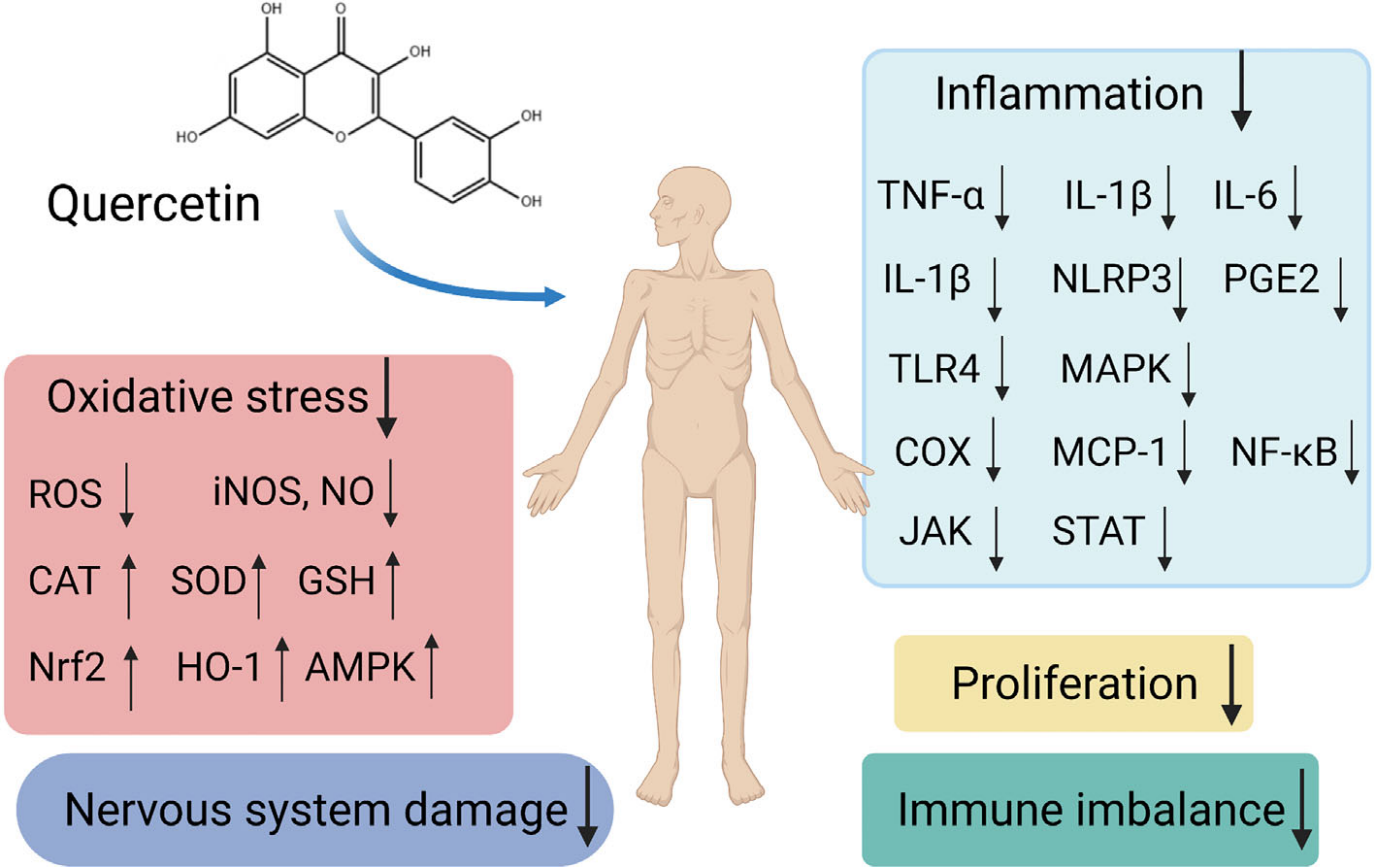
Schematic representation of different signaling pathways and targets by quercetin as a potential therapeutic strategy in autoimmune diseases.

Autoimmune diseases are systemic conditions that are difficult to cure, and patients often require long-term treatment. The development and pathogenesis of autoimmune diseases involve multiple associations and interacting factors. The diversity and complexity of associations between the components involved are likely to limit the effects of therapies, and contribute to adverse side effects. Quercetin possesses anti-inflammatory, anti-oxidant, neuroprotective, and anti-allergic activities, as well as the capacity to interact with multiple molecules and targets (**Figure 1**). Moreover, treatments involving appreciable doses of quercetin have evidently been low-toxic or non-toxic. Quercetin will be expected to become a potential opportunity and supplement for the treatment and prevention of autoimmune diseases. However, it is particularly important since there is no evidence so far that quercetin could reduce the morbidity and mortality of autoimmune diseases. The direct effects of quercetin on immune imbalance in patients are still unconfirmed. Further clinical studies are still lacking, most of the underlying mechanisms have been reported in animal models and need to be demonstrated for their pharmacological application. It is necessary to study it thoroughly with regard to high doses in order to detect possible undesirable side effects. Only well-designed randomized controlled trials with large sample sizes will reveal the biosafety and efficacy of these currently experimental applications. Due to its poor aqueous solubility, high metabolic rate, poor oral bioavailability and absorption, and rapid body clearance, its application is limited. A better knowledge about the pharmacodynamics, pharmacokinetics, and enhanced bioavailability of quercetin are also necessary. In view of future directions and priorities, we suggest some protocol recommendations for future studies. Scholars can explore different routes of administration under the different medical conditions of different diseases such as the local injection for anti-inflammatory effects in RA. In *in vitro* studies, three-dimensional co-cultures of different cells that are responsible for a disease can mimic the microenvironment. For instance, fibroblast synovial cells, osteoblasts, and osteoclasts can be used in the studies of RA, and intestinal epithelial cells and fibroblasts can be adopted in IBD studies. Finally, it is valuable to increase the solubility, bioavailability, and target specificity of quercetin inside the human body. Investigating structurally relevant compounds of quercetin presents a novel subject for further experiments.

## Author Contributions

PS wrote the first draft of the manuscript. All authors contributed to the article and approved the submitted version.

## Conflict of Interest

The authors declare that the research was conducted in the absence of any commercial or financial relationships that could be construed as a potential conflict of interest.
